# Rod responses produce the peripheral flicker illusion

**DOI:** 10.1177/20416695251333732

**Published:** 2025-04-23

**Authors:** Meidi Niikawa, Hiroyuki Ito

**Affiliations:** Department of Media Design, Kyushu University, Japan

**Keywords:** peripheral flicker illusion, color, peripheral vision, rod-cone interaction

## Abstract

When a green/blue object is presented on a red background and viewed in peripheral vision, the object is seen to flash twice or to flicker (the peripheral flicker illusion). We showed that the ratio of photopic luminances of the object and the red background determines the optimal photopic luminance of the green/blue object required for the illusion to occur. The results were analyzed using scotopic luminance to investigate the role of rod responses. It was found that the scotopic luminance of the green/blue object should be higher than that of the red background for the illusion to occur. This suggests that the red background enhances the flickering impression of the object when there is a sudden increase in the rod responses.

## How to cite this article

Niikawa, M., & Ito, H. (2025). Rod responses produce the peripheral flicker illusion. *i-Perception*, *16*(0), 1–11. https://doi.org/10.1177/20416695251333732

## Introduction

**Figure 1.**
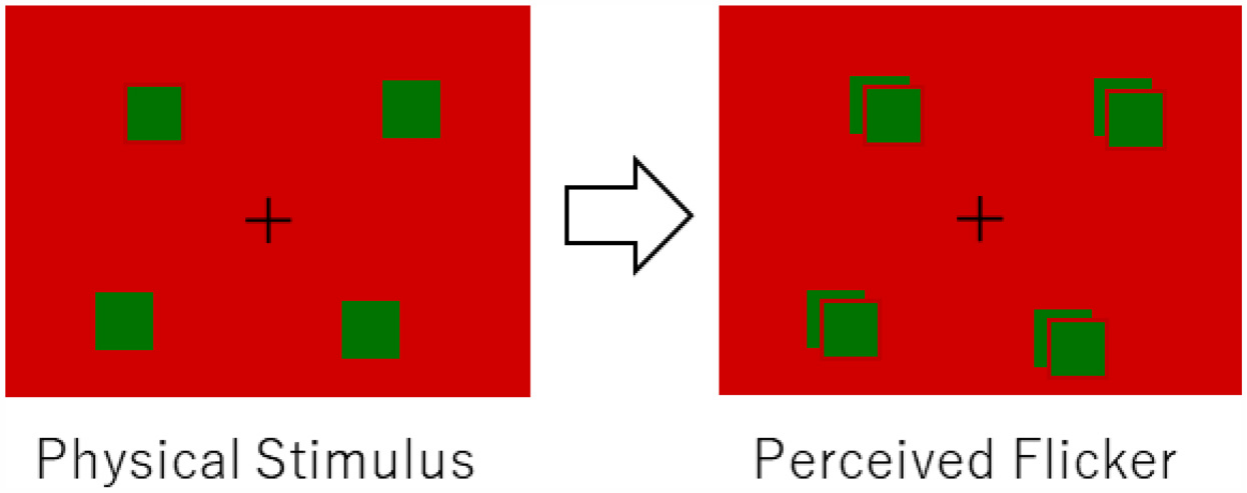
*Peripheral flicker illusion*. When green/blue objects appear on a red background, they seem to flash twice or flicker. Please try Video 1 in a darkened environment. This movie can also be viewed on YouTube: https://youtu.be/5Dxg64TUqUo.

There are some illusions reported to date, where a single visual flash is perceived as double or triple flashes (e.g., Wilson & Singer, 1981; Chatterjee et al., 2011; Csibri et al., 2014). These illusions are observed when a single test flash is presented with physical multiple flashes of visual or auditory stimuli. Ito and Koizumi (2017) found that when a green/blue object appeared in a peripheral visual field, the object was seen to flash twice or flicker (the *peripheral flicker illusion*) as shown in Figure 1 (try the demonstration movie “Video 1”). They found that, to produce a strong effect, the object color should be green/blue (not red), the background color should be red (not blue or green), the photopic luminance of the object should be lower than the photopic luminance of the background, the display should be viewed in mesopic vision, and the object should appear in peripheral vision. From the restricting conditions for the illusion to occur, they inferred that the illusion would be related to peripheral mechanisms (e.g., rod responses), not to the central process that integrates multisensory signals as in the sound-induced visual flash phenomenon (Shams et al., 2000; Roseboom et al., 2013). As a tentative hypothesis, they proposed that three components might constitute the perceptual flicker illusion: (1) the rise of cone responses elicited by the appearance of a new object, (2) the delayed rise of rod responses elicited also by the object appearance, and (3) the fall of cone responses caused by the red background disappearance due to the object appearance. Each of the three components may be accompanied by some overshoot.

The purpose of the present study was to investigate the relationship between the object and the red-background luminances (photopic or scotopic) necessary to produce the illusion. The tentative hypothesis noted above assumed that when a green/blue object appears, rod responses rise. This assumption requires a condition where the rod response to the appearing object is larger than that to the red background that disappears when the object appears. Therefore, we examined whether the illusion arose only when the scotopic luminance of the object was higher than that of the red background. In Ito and Koizumi (2017), it was not obvious whether the strength of the illusion was determined by the relationship between the photopic luminance of the object and the background or by the photopic luminance of the object alone. We tested the effect of photopic or scotopic luminance relationships between the object and the red background on the *peripheral flicker illusion*.

## Method

### Observers

Five males and one female participated. Five of them were naïve observers aged 22–26 years. The other was the second author, in his early 60s. All had normal or corrected-to-normal trichromatic vision. Informed consent was obtained.

### Apparatus and Stimuli

All the stimuli were created by a computer (Dell, Inspiron 400) and displayed on an organic light-emitting diode display (Sony, PVM-2541, 60 Hz). Detailed temporal characteristics of the display were described in Ito et al. (2013). CIE 1931 xy chromaticity values were (0.6766, 0.3230) for red, (0.1877, 0.7239) for green, (0.1417, 0.0500) for blue, and (0.3117, 0.3229) for gray. The screen displayed a 1920 (horizontal) × 1080 (vertical) pixel matrix.

**Figure 2.**
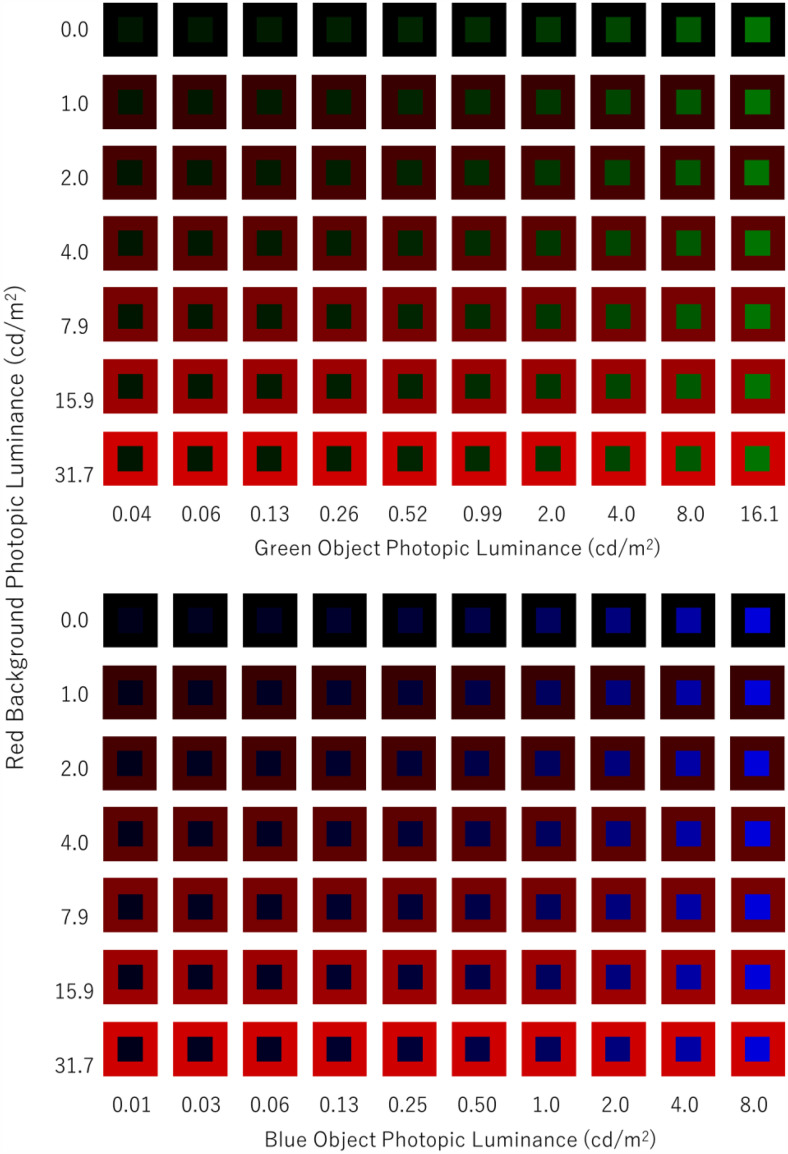
Color combinations tested in experiment 1.

The tested combinations of object and background colors are displayed in Figure 2. The luminance of the red background was varied in seven levels, i.e., 0.0, 1.0, 2.0, 4.0, 7.9, 15.9, or 31.7 photopic cd/m^2^. The background at 0.0 cd/m^2^ means a black background. The tested object colors were green and blue. The luminance of the green object was varied in 10 steps, i.e., 0.04, 0.06, 0.13, 0.26, 0.52, 0.99, 2.0, 4.0, 8.0, or 16.1 photopic cd/m^2^. The luminance of the blue object was also varied in 10 steps, i.e., 0.01, 0.03, 0.06, 0.13, 0.25, 0.50, 1.0, 2.0, 4.0, or 8.0 photopic cd/m^2^.

As a reference for flicker evaluation, a gray area was presented at the beginning of each trial. Both the colored objects and the reference object were squares of 100 pixels per side subtending 2.7° of visual angle. A fixation cross was placed in the center of the screen.

**Figure 3.**
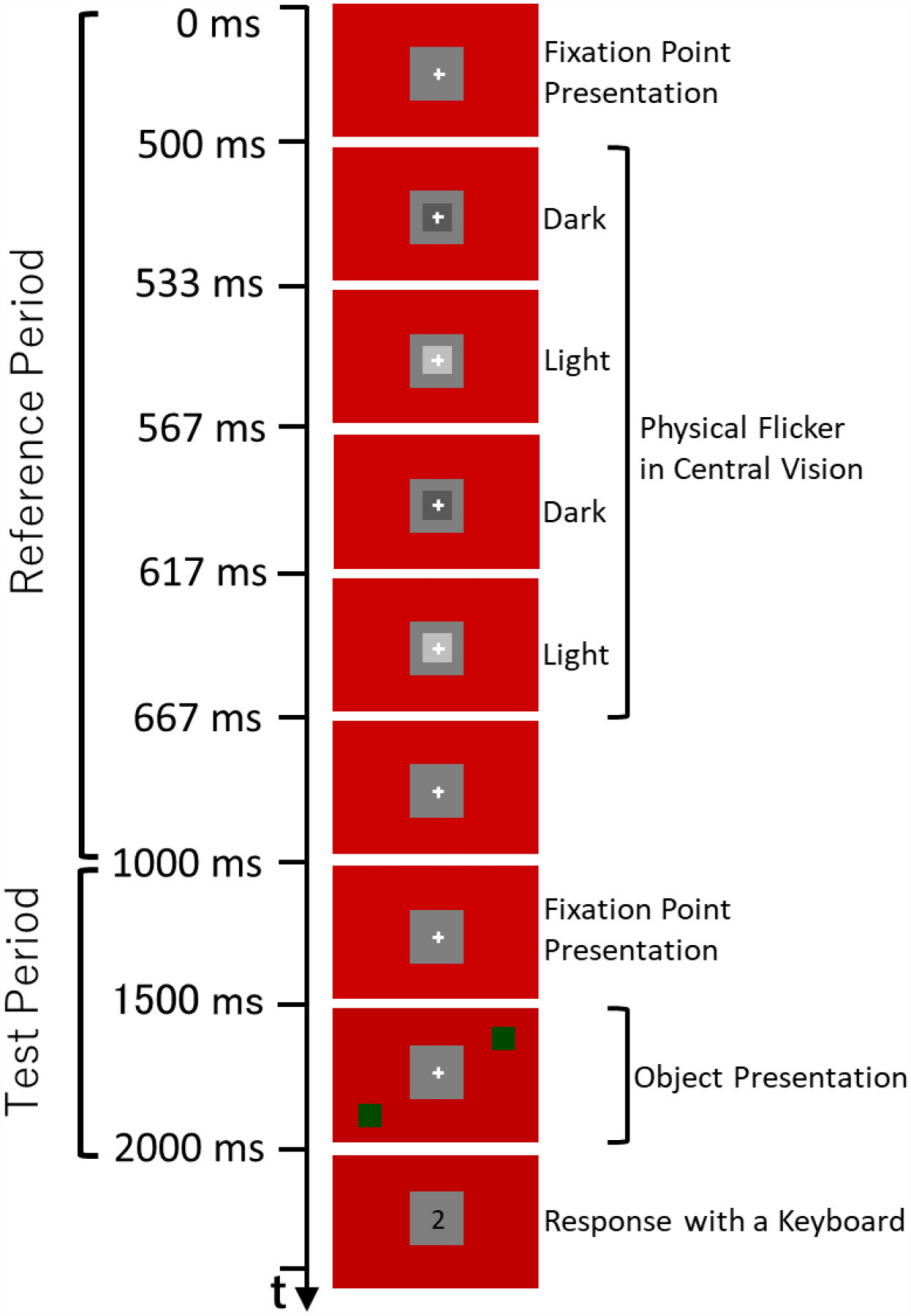
Time course of a trial sequence (see details in the text).

The tested objects were presented in lower left and upper right positions from the fixation point (26.6° orientation from the horizontal, see Figure 3), or in upper left and lower right positions. The center of each object was 11.9° from the center of the fixation cross. This spatial configuration was consistent with Ito and Koizumi. (2017).

### Procedure

Observers viewed the display at a distance of 60 cm in a darkened room. They adapted to the experimental environment during a training session (for about 10 min). A dark adaptation period was not used because they perceived the illusion within a few minutes of entering the laboratory and because the transients of the stimuli were sometimes intense and felt uncomfortable for dark-adapted eyes. Their heads were restricted by a chinrest. As shown in Figure 3, one trial consisted of the reference flicker presentation period and the test object presentation period. In the reference period, first, a red background was presented with a white fixation cross (16.1 photopic cd/m^2^) in a gray square (6.0 photopic cd/m^2^) placed in the center of the screen. The fixation cross remained visible throughout the trial. After 500 ms, a physical flicker was presented for 167 ms as a reference stimulus. That is, a dark gray object (4.9 photopic cd/m^2^) appeared in the center and was displayed for two frames (33.3 ms), then a light gray object (7.1 photopic cd/m^2^) appeared and displayed for two frames (33.3 ms). The sequence of the dark gray and light gray object presentation was repeated again with a 50-ms duration for each gray object. It is known that a gray object appearing in central vision on a gray background does not cause illusory flicker (Ito & Koizumi, 2017). The physical flicker sequence followed Ito and Koizumi (2017), which mimicked the subjective flicker.

After an interval of 333 ms, the test period started. First, a red background was presented for 500 ms, followed by the green/blue object presentation for 500 ms. Observers evaluated the flicker impression of the green/blue object relative to that of the reference stimulus. Observers assigned a value of 10 if the flicker appeared to be the same strength as that of the reference stimulus, a value of 0 if no flicker was perceived, and intermediate values between 1 and 9 or above 10 to represent perceived flicker that was weaker or stronger than the reference, respectively. Observers were not instructed to focus on any particular attribute, such as duration, amplitude, or frequency. They input their estimated values using a computer keyboard.

Two object color (green/blue) conditions and seven background photopic luminance conditions were measured in separate sessions. One experimental session included 50 trials (10 object photopic luminance conditions with five repetitions for each). Two sessions were conducted for each of 14 combined conditions (two object color with seven background photopic luminances). Thus, in total 1,400 trials (two object color × 10 object photopic luminance × seven background photopic luminance × 10 times repetitions) were conducted. Stimulus order was randomized within a session. The order of sessions was also randomized.

## Results and Discussion

**Figure 4.**
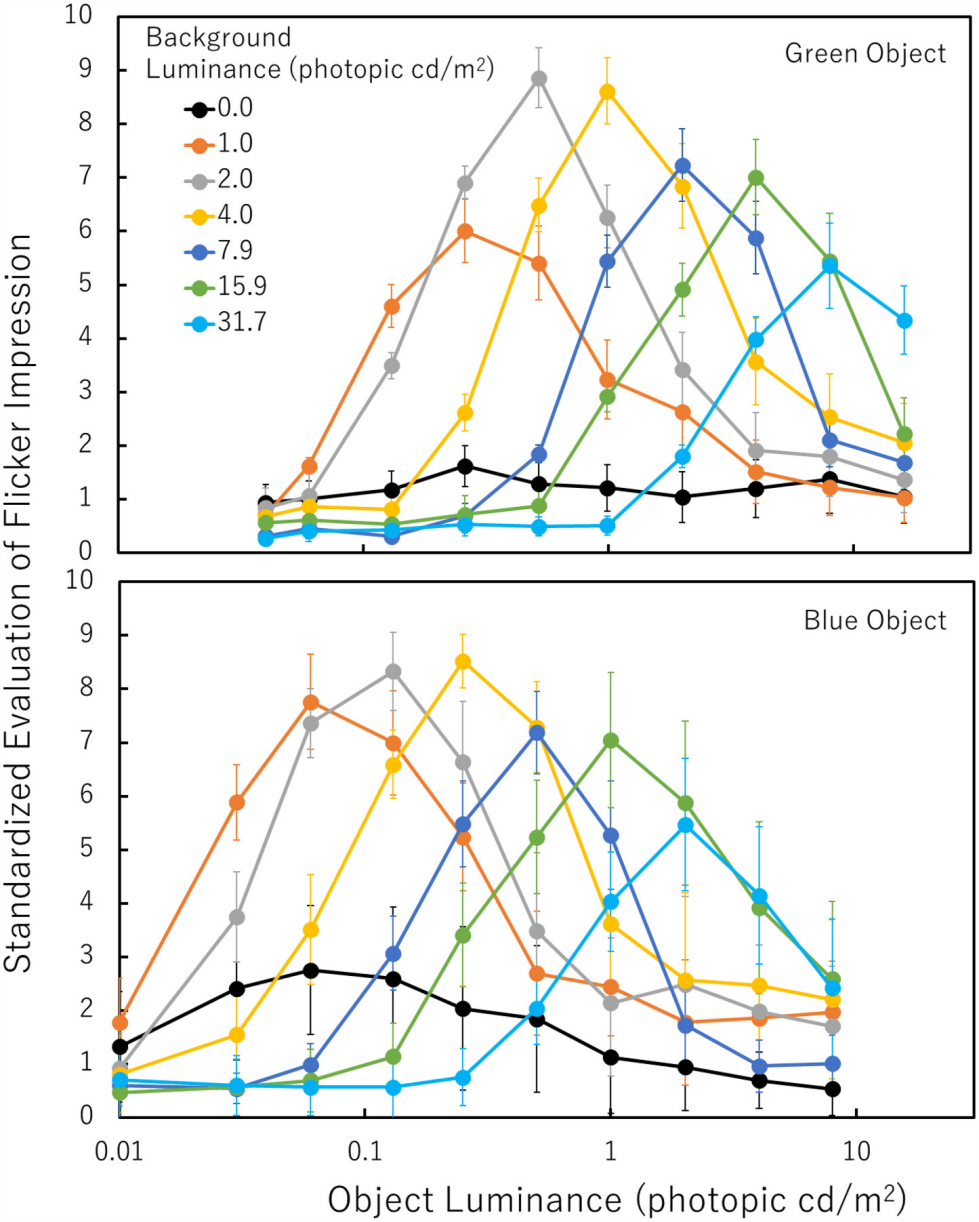
Standardized evaluation of the flicker impressions of a green object (top panel) or a blue object (bottom panel) under varied combinations of object and background photopic luminances. Error bars indicate SEs.

Figure 4 shows the results for the green/blue object conditions. The individual differences in evaluated values were large although the trend was similar. One reason for the individual difference could be the lack of a sufficient dark adaptation period. Another reason could be the different response standards of each observer. To equalize the contribution of each observer’s evaluation, the values in Figure 4 were individually standardized in the way that the highest value within each observer's evaluation for each object color was 10. The raw value of the highest evaluation from each of the observers was 6.4, 5.5, 5.3, 4.9, 2.7, or 2.6 for the green object and 5.7, 5.1, 5.0, 4.7, 2.2, or 2.1 for the blue object.

As shown in Figure 4, top panel, it is clear that the evaluated illusion strength for a green object under each background photopic luminance condition produced a kind of *bell curves* as the photopic luminance of the object increased, except that under the 0.0 photopic cd/m^2^ (black) background condition. As for the black background condition, the photopic luminance of the tested object was always higher than that of the black background. The object with a black background produced a weak illusion and did not show a clear peak in the illusory effect in the varied object photopic luminance. Although the existence of the red background should not be a necessary condition to produce the illusion, it is evident that the red background greatly enhanced the illusory flicker. When the red background luminance was 2.0 or 4.0 photopic cd/m^2^, the evaluation was highest. We found a significant effect of the red background photopic luminance on the highest evaluated values (*F* (6, 30) = 7.085, *p *= .0001, *ηp^2 ^*= 0.586). The black background condition produced a weaker illusion than the 1.0, 2.0, 4.0, 7.9, 15.9, or 31.7 photopic cd/m^2^ background condition (Ryan's method, *p *< .05). The photopic luminance of the object with the highest evaluated value increased as the photopic luminance of the red background increased. That is, in Figure 4, the peak position shifts rightward according to the increase in background photopic luminance. We found a significant effect of the background photopic luminance on the best object photopic luminance through logarithmic transformation (*F*(6, 30) = 22.547, *p *< .0001, *ηp^2 ^*= 0.818). With the object photopic luminance steps tested here, the best green-object photopic luminance seems to be constant at around 1/4 in the object/background photopic luminance ratio except for the black background condition. For example, when the red-background luminance was photopic 4.0 cd/m^2^, the best green-object luminance was around 1.0 photopic cd/m^2^.

As shown in Figure 4, bottom panel, it is clear that the evaluated illusion strength for a blue object under each background photopic luminance condition also produced a kind of *bell curves* as the object photopic luminance increased. In the black background condition, moderate flicker impressions were reported under very low (around 0.1 photopic cd/m^2^) object luminance conditions. As in the green object conditions, although the presence of a red background is not a necessary condition to produce the illusion, especially for a dark blue object, it is evident that the red background greatly enhanced the illusory flicker. The basic effect of the illusion might be produced by the dissociation of the rise of rod and cone responses and that the red background enhanced this effect.

We conducted statistical tests to compare the highest values among the seven background photopic luminance conditions. The effect of the background photopic luminance on the highest evaluated values was significant (*F*(6, 30) = 4.166, *p *= 0.0037, *ηp^2 ^*= .455). The highest values under the black background condition were significantly lower than those under the 1.0, 2.0, or 4.0 photopic cd/m^2^ background condition (*p *< .05). The photopic luminance of the object with the highest evaluated value increased as the photopic luminance of the red background increased. We found a significant effect of the background photopic luminance on the photopic luminance of the best object condition through logarithmic transformation (*F*(6, 30) = 17.664, *p *< .0001, *ηp^2 ^*= .779). With the object photopic luminance steps used here, the best blue-object photopic luminance seems to be constant at around 1/16 in the object/background photopic luminance ratio except for the black background condition. For example, when the red background luminance was 4.0 photopic cd/m^2^, the best blue-object luminance was around 0.25 photopic cd/m^2^. This result is consistent with Ito and Koizumi (2017) showing that the photopic luminance of the best object condition was below the background photopic luminance and lower for the blue object than for the green object. It is a new finding that the ratio between the object and background photopic luminances, not the object photopic luminance, was a determinant of the illusion strength.

**Figure 5.**
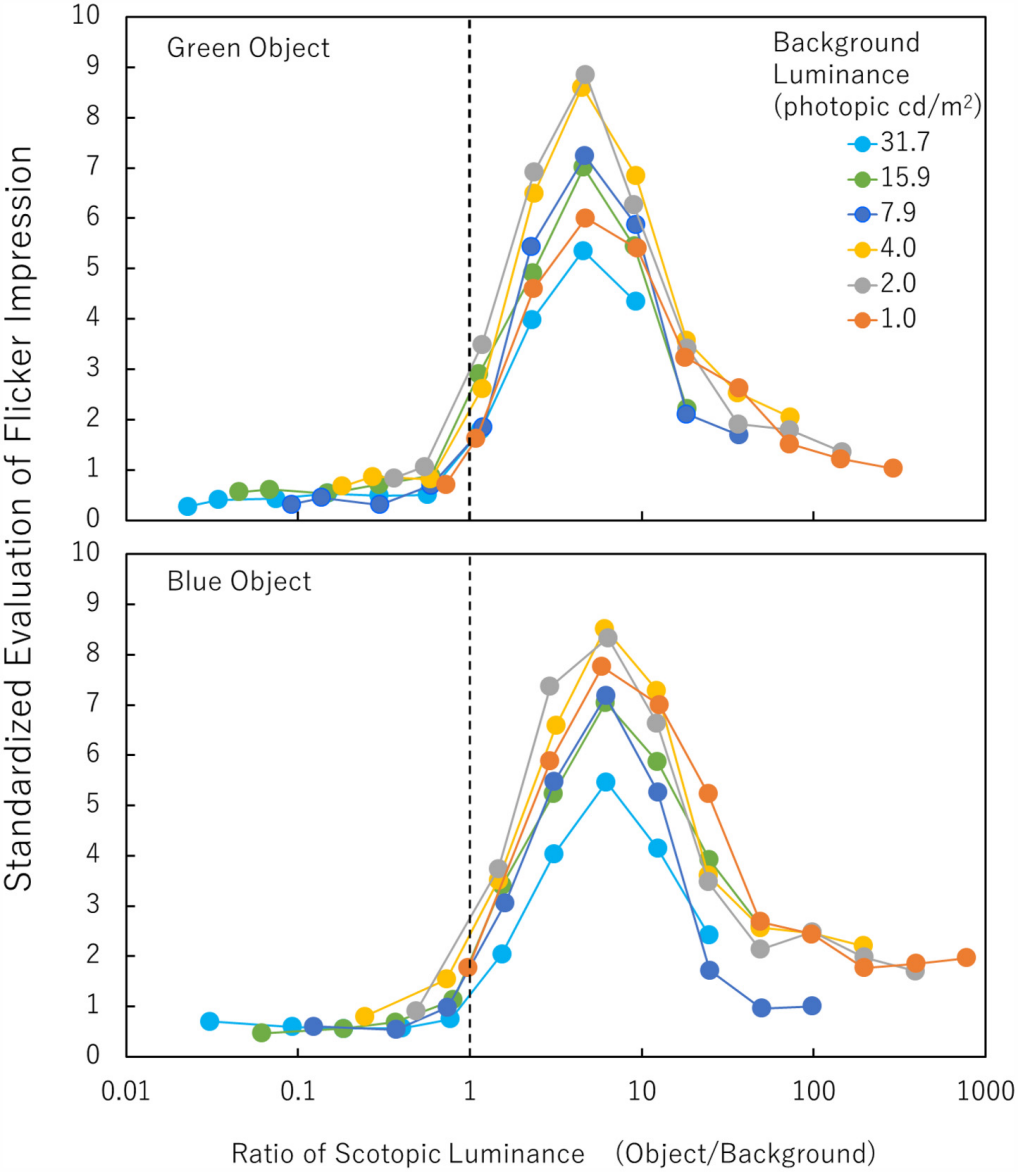
Standardized evaluation of flicker impression as a function of the ratio of the object scotopic luminance to the background scotopic luminance (see details in the text).

To confirm the role of rod responses, the results were reanalyzed, using values in scotopic luminance. Scotopic luminances of the present stimuli were measured with a radiometer (International Light Technology Inc, ILT5000, equipped with a scotopic luminance detector (SED033/ZCIE/R)). While rods are highly sensitive to blue light, their sensitivity to red light is low. For example, while red in 31.7 photopic cd/m^2^ on our display corresponded to 4.7 scotopic cd/m^2^, green in 8.0 photopic cd/m^2^ or blue in photopic 2.0 cd/m^2^ corresponded to 21.2 scotopic cd/m^2^ or 28.8 scotopic cd/m^2^, respectively. As noted earlier, if the rise of the rod response is needed for the illusion, the rod response to the appearing object should be larger than that to the disappearing red background to cause the illusion. Figure 5 shows the evaluated illusion strength as a function of the ratio between the scotopic luminance of the object and the scotopic luminance of the red background. The value of 1.0 means that the scotopic luminance of the object equaled to that of the red background. When the ratio is above 1.0, the scotopic luminance of the object is higher than that of the red background. As clearly seen in Figure 5, irrespective of the photopic luminance of the red background and irrespective of the object colors, when the ratio was below 1.0 (i.e., the scotopic luminance of the object was lower than that of the red background), the evaluated illusion strength was very low. When the ratio is above 1.0, the evaluated illusion strength steeply becomes high. We think that this tendency is an evidence showing that the rise of the rod responses at the object appearance is one of the main components constituting this illusion. When the ratio was 4.5 or 6.1 for a green or blue object, respectively, the evaluated value was the highest. The two values were virtually the same because the difference was below the step size of the photopic luminance settings of the object. When the ratio is over 10, the evaluated illusion strength decreases. We think that when the photopic luminance or the scotopic luminance of the red background is too low relative to that of the object, the effect of the red light is not enough to produce a strong illusion for the bright objects. One may argue that the drops in illusion strength at high object-to-background scotopic luminance ratios are related to the saturation of rod responses. However, as shown in Figure 4, the drops in illusion strength were determined not by the object (scotopic) luminance itself but by the ratio between the object and background (scotopic) luminances. These results indicate that the rise of the rod response to the appearing object is one of the main components of the illusion and that the red background does enhance the illusory effect.

When the luminance of the red background was highest within the range tested here (photopic 31.7 cd/m^2^), the illusion strength seemed to decrease although the difference was not statistically significant. One possible reason is that as the red background elicits lots of rod responses, it is difficult that the presented object elicits further rod responses due to rod saturation. However, when the object appeared, the red background vanished. Thus, the effect of rod saturation may appear depending not on the scotopic luminance of the red background but only on the scotopic luminance of the object. There has been debate on the luminance level where the rod saturates. Although it is generally believed that rods saturate in daylight, some research showed that the rods responded well in the photopic range (Aguilar & Stiles, 1954; Tikidji-Hamburyan et al., 2017).

Some illusions are known to occur with a blue object on a red background or the opposite combination, e.g., the fluttering-heart illusion (Helmholtz, 1867/1962). The reasons for the illusion were proposed as a difference in response latency to each color (Helmholtz, 1867/1962), a difference in response latencies of the rods and cones (von Kries, 1896), lateral interactions between the rods and cones at the object border (von Grünau, 1975, 1976), a differential in the perceived velocities of chromatic and achromatic motion (Nguyen-Tri & Faubert, 2003), a difference in latency between high and low contrast borders (Kitaoka & Ashida, 2007), and reversed phi produced by fast cone responses and delayed rod responses (Anstis & Macleod, 2015). Some of the above-proposed reasons have common features with our hypothesis for the *peripheral flicker illusion* in the assumption that the illusions are based on the difference in response latency between the rods and cones and on the lateral interaction of the rods and cones at the object border. Although our stimuli did not move as that for the fluttering-heart illusion, the leading edge of the moving blue “heart” could be interpreted as producing a situation of the sudden appearance of a blue object on a red background on the retina. Thus, it may be useful to study the relationship between them.

It is known that there are two rod pathways, fast and slow (see review in Stockman & Sharpe, 2006). The delay of rod signals relative to cone signals is reported to be 33.3 ms for the fast pathway and 66.7 ms for the slow pathway. It is unclear which pathway might be involved in the present illusion. The task of matching the onset time of a red object used as a probe with the perceived onset time of the present illusion produced by a blue object may indicate the latency of rods in this illusion. Another possibility is that the perceived flicker frequency reflects the temporal resolution of the rod pathway involved. However, it is also known that rod spectral sensitivity is held constant from the bottom (initial quantum capture) to the top (conscious level) (Baylor et al., 1984). Therefore, one might argue that the interaction between rod and cone properties could occur anywhere along the visual pathway. By testing a dichoptic presentation of the stimuli, we can at least confirm whether or not the process responsible is post-cortical.

From another point of view, it has been often suggested that a red background suppresses the magnocellular pathway. The malfunction of the magnocellular pathways or suppression of the magnocellular pathways by adaptation to a red background is sometimes related to research on dyslexia (Skottun, 2000, as a review), metacontrast masking (Williams et al., 1991), or motion perception (Chapman et al., 2004) while it may not be true that the red background only suppresses the magnocellular pathway, not affecting the parvocellular pathway (Skottun, 2004). In addition, Cormenzana Mendez et al. (2022) suggested that rod signals suppress the luminance pathway while they contribute to color perception in mesopic vision. If a red background or rod signals differently affect the activities of the magnocellular and parvocellular pathways, a failure in the signal integration of object appearance from both pathways could occur.

## Conclusion

In this study, we showed that the photopic luminance of the object required for the illusion to occur was proportionate to the photopic luminance of the red background. In terms of scotopic luminance, to produce a strong illusion, the scotopic luminance of the object should be higher than that of the red background, irrespective of the object colors and background photopic luminances. This suggests that the sudden increase in rod responses when an object appears on a red background is a critical factor in the *peripheral flicker illusion*.

## Supplemental Material

**Figure:** Video 1.

## References

[bibr1-20416695251333732] Aguilar M. Stiles W. S. (1954). Saturation of the rod mechanism of the retina at high levels of stimulation. Optica Acta: International Journal of Optics, 1(1), 59–65. 10.1080/713818657

[bibr2-20416695251333732] Anstis S. Macleod D. (2015). Why hearts flutter: Distorted dim motions. Journal of Vision, 15(3), 23. https://doi.org/10.1167/15.3.23 PMC4374759 25814549

[bibr3-20416695251333732] Baylor D. A. Nunn B. J. Schnapf J. L. (1984). The photocurrent, noise and spectral sensitivity of rods of the monkey macaca fascicularis. Journal of Physiology, 357, 575–607. 10.1113/jphysiol.1984.sp015518 6512705 PMC1193276

[bibr4-20416695251333732] Chapman C. Hoag R. Giaschi D. (2004). The effect of disrupting the human magnocellular pathway on global motion perception. Vision Research, 44(22), 2551–2557. 10.1016/j.visres.2004.06.003 15358070

[bibr5-20416695251333732] Chatterjee G. Wu D-A. Sheth B. R. (2011). Phantom flashes caused by interactions across visual space. Journal of Vision, 11(2), 14, 1–17. 10.1167/11.2.14 21343326

[bibr6-20416695251333732] Cormenzana Méndez I. Martín A. O’Donell B. (2022). Temporal integration of rod signals in luminance and chromatic pathways. Journal of the Optical Society of America, A, 39(10), 1782–1793. 10.1364/JOSAA.462581 36215550

[bibr7-20416695251333732] Csibri P. Kaposvári P. Sáry G. (2014). Illusory flashes and perception. Journal of Vision, 14(3), 6. https://doi.org/10.1167/14.3.6 24599944

[bibr8-20416695251333732] Helmholtz H. (1962). Treatise on physiological optics (Vol. 2) ( Southall J. P. C. Trans.). Dover. (Reprinted from Handbuch der physiologischen optic, 1867, Leipzig: Voss.

[bibr9-20416695251333732] Ito H. Koizumi T. (2017) The Peripheral Flicker Illusion. i-Perception. December, 1–13. 10.1177/2041669517747891 PMC5761921 29344331

[bibr10-20416695251333732] Ito H. Ogawa M. Sunaga S. (2013). Evaluation of an organic light-emitting diode display for precise visual stimulation. Journal of Vision, 13(7), 6. 10.1167/13.7.6 23757510

[bibr11-20416695251333732] Kitaoka A. Ashida H. (2007). A variant of the anomalous motion illusion based upon contrast and visual latency. Perception, 36(7), 1019–1035. 10.1068/p5362 17844967

[bibr51-20416695251333732] Nguyen-Tri D. Faubert J. (2003). The fluttering-heart illusion: A new hypothesis. Perception, 32(5), 627–634. 10.1068/p3228 12854649

[bibr12-20416695251333732] Roseboom W. Kawabe T. Nishida S. (2013). The cross-modal double flash illusion depends on featural similarity between cross-modal inducers. Scientific Reports, 3, 3437. 10.1038/srep03437 24310546 PMC3853685

[bibr13-20416695251333732] Shams L. Kamitani Y. Shimojo S. (2000). Illusions: What you see is what you hear. Nature, 408(6814), 788. 10.1038/35048669 11130706

[bibr14-20416695251333732] Skottun B. C. (2000). The magnocellular deficit theory of dyslexia: The evidence from contrast sensitivity. Vision Research, 40(1), 111–127. 10.1016/S0042-6989(99)00170-4 10768046

[bibr15-20416695251333732] Skottun B. C. (2004). On the use of red stimuli to isolate magnocellular responses in psychophysical experiments: A perspective. Visual Neuroscience, 21(1), 63–68. https://doi.org/10.10170S0952523807041063 15137582 10.1017/s0952523804041069

[bibr16-20416695251333732] Stockman A. Sharpe L. T. (2006). Into the twilight zone: The complexities of mesopic vision and luminous efficiency. Ophthalmic and Physiological Optics, 26(3), 225–239. 10.1111/j.1475-1313.2006.00325.x 16684149

[bibr17-20416695251333732] Tikidji-Hamburyan A. Reinhard K. Storchi R. Dietter J. Seitter H. Davis K. E. Idrees S. Mutter M. Walmsley L. Bedford R. A. Ueffing M. Ala-Laurila P. Brown T. M. Lucas R. J. Münch T. A. , (2017). Rods progressively escape saturation to drive visual responses in daylight conditions. Nature Communications, 8(1), 1813. 10.1038/s41467-017-01816-6 PMC5703729 29180667

[bibr18-20416695251333732] von Grünau M. W. (1975). The “fluttering heart” and spatio-temporal characteristics of color processing—II. Lateral interactions across the chromatic border. Vision Research, 15, 437–440. 10.1016/0042-6989(75)90095-4 1136163

[bibr19-20416695251333732] von Grünau M. W. (1976). The “fluttering heart” and spatio-temporal characteristics of color processing—III. Interactions between the systems of the rods and the long-wavelength cones. Vision Research, 16, 397–401. 10.1016/0042-6989(76)90203-0 941416

[bibr20-20416695251333732] von Kries J. (1896). Uber die wirkung kurzdauernder lichtreize auf das sehorgan [translation: On the effect of brief light stimuli upon the eye]. Zeitschrift für Psychologie und Physiologie der Sinnesorgane, 12, 81–101.

[bibr21-20416695251333732] Williams M. C. Breitmeyer B. G. Lovegrove W. J. Gutierrez C. (1991). Metacontrast with masks varying in spatial frequency and wavelength. Vision Research, 31(11), 2017–2023. 10.1016/0042-6989(91)90196-C 1771785

[bibr22-20416695251333732] Wilson J. T. L. Singer W. (1981). Simultaneous visual events show a long-range spatial interaction. Perception & Psychophysics, 30, 107–113. 10.3758/BF03204467 7301509

